# Including Classical Galactosaemia in the Expanded Newborn Screening Panel Using Tandem Mass Spectrometry for Galactose-1-Phosphate

**DOI:** 10.3390/ijns5020019

**Published:** 2019-05-04

**Authors:** Arieh S. Cohen, Marta Baurek, Allan M. Lund, Morten Dunø, David M. Hougaard

**Affiliations:** 1 Danish Center for Newborn Screening, Statens Serum Institut, 2300 Copenhagen, Denmark; 2 Centre for Inherited Metabolic Diseases, Departments of Paediatrics and Clinical Genetics, Copenhagen University Hospital, 2100 Copenhagen, Denmark

**Keywords:** galactosaemia, mass spectrometry, newborn screening, GALT

## Abstract

Galactosaemia has been included in various newborn screening programs since 1963. Several methods are used for screening; however, the predominant methods used today are based on the determination of either galactose-1-phosphate uridyltransferase (GALT) activity or the concentration of total galactose. These methods cannot be multiplexed and therefore require one full punch per sample. Since the introduction of mass spectrometry in newborn screening, many diseases have been included in newborn screening programs. Here, we present a method for including classical galactosaemia in an expanded newborn screening panel based on the specific determination of galactose-1-phosphate by tandem mass spectrometry. The existing workflow only needs minor adjustments, and it can be run on the tandem mass spectrometers in routine use. Furthermore, compared to the currently used methods, this novel method has a superior screening performance, producing significantly fewer false positive results. We present data from 5500 routine newborn screening samples from the Danish Neonatal Screening Biobank. The cohort was enriched by including 14 confirmed galactosaemia positive samples and 10 samples positive for other metabolic disorders diagnosed through the Danish newborn screening program. All galactosaemia positive samples were identified by the method with no false positives. Furthermore, the screening performance for other metabolic disorders was unaffected.

## 1. Introduction

Classical galactosaemia is an inborn error of galactose metabolism. It is an autosomal recessive disease, which is caused by a deficiency in the GALT enzyme. This enzyme converts galactose-1-phosphate (GAL-1-P) to glucose-1-phopshate. This leads to an inability to metabolize galactose, which in turn causes a build- up of GAL-1-P and other galactose metabolites. Classical galactosemia is a lethal condition if untreated. Although the precise mechanism of the disease is not fully understood, it typically presents with cataracts, liver failure and a propensity for *E. coli* sepsis. The incidence of classical galactosemia is reported to be 1:30,000 to 1:100,000 live births depending on the population studied [1,2,3]. The condition is notably prevalent in the traveler population of Ireland with an incidence of 1:430 [4]. The only treatment available to date is a galactose-restricted diet. In the newborn phase, the main source of galactose is from the lactose in milk. It is thus important to immediately replace breast milk or formula based on cow milk with other formulae such as soy milk based formula [5].

Galactosaemia has been part of many newborn screening programs for several decades. All states in the USA screen for the disease and several European countries screen for it as well. Despite the long history of newborn screening for the disease, there is some controversy to the suitability of including it in the newborn screening panel. There are primarily two issues that underpin this controversy. The first is the long-term outcome including issues concerning cognitive decline and neurological manifestations. Among those that develop these symptoms, there is no evidence that the long-term outcome is improved by detecting the disease before the presentation of clinical symptoms [2]. However, some patients do not develop such symptoms. In addition, it is a life-threatening disease, and early detection and treatment can prevent serious complications such as liver failure, neurologic complications such as cerebral haemorhage and kernicterus, as well as E. Coli sepsis. The second issue is the poor screening performance that has been observed in many programs. The positive predictive value (PPV) of the current tests in use has been reported to be between 0.9% to 64.3% (average 8.1%) [2]. The large number of false positives are a burden for the health care system and for the families that must undergo further investigation [2,6].

Galactosaemia most often presents within the first weeks after birth. Initial symptoms include poor feeding, vomiting, diarrhea, lethargy and hypotonia. In order for screening to be of value for the patients, it is necessary to achieve rapid, reliable screening results. Preferably, the positive outcomes should be known within the first week of life. There are several diseases included in expanded newborn screening programs that put similar demands on the screening method (e.g., maple syrup urinary disease (MSUD) [7]).

The two main types of assay used for galactosaemia screening are enzyme activity tests and total galactose tests. Many programs utilize both types of test in a two-tier set-up. Both assays have advantages and disadvantages. By testing GALT activity, the actual mechanism of the disease is evaluated. Low enzyme activity is highly indicative of galactosaemia. However, enzyme activity is sensitive to handling and transport. High temperature and humidity is detrimental to enzyme activity in newborn screening samples and can lead to false positive samples. Total galactose assays do not call for long incubation times and thus are more rapid than enzyme activity assays. However, these assays are not as specific as they do not differentiate between GAL-1-P, galactose and UDP galactose.

Tandem mass spectrometry (TMS) based screening for galactosemia has previously been reported [8,9,10] as a stand-alone method, which is not multiplexed with other analyses. It is based on measuring the content of hexose mono-phosphates (HMP) in the newborn screening sample. In this instance, the actual biomarker for the disease is GAL-1-P. However, it was not possible to differentiate between various HMPs without chromatographic separation. The rationale of measuring total HMP is that elevated levels of GAL-1-P will cause total HMP to be elevated and thus provide a positive screening result. In the method we present we overcome this limitation by selectively depleting interfering HMPs using a mild hydrazine solution. In this manner, improved sensitivity and selectivity is achieved.

The fact that screening for galactosaemia was not multiplexed with the expanded newborn screening panel was due to technical limitations in mass spectrometers used at that time. The instruments available in 2001 were far less sensitive than the instruments that are on the market today. In order to achieve the necessary sensitivity, newborn screening samples were derivatized with butanol. The derivatization rendered the analytes far more volatile, which provided increased sensitivity in electrospray tandem mass spectrometry (TMS). However, GAL-1-P was not stable during this derivatization and could thus not been multiplexed into the screening with this approach. Previously, instruments have not been able to switch polarity during a run. Expanded newborn screening is run in positive mode TMS. However, the phosphate group in GAL-1-P is permanently negatively charged and must be detected in negative mode. Thus, it was not possible to multiplex GAL-1-P measurement into the newborn screening program even if done non-derviatized.

With the advent of more sensitive TMS instruments it has become more common to run non-derivatized expanded newborn screening. Instruments have also improved with regards to polarity switching. Thus with modern instruments there are no technical reasons for not including galactosaemia in the expanded newborn screening panel.

Here, we present a method for including galactosaemia screening into the tandem mass spectrometry expanded newborn screening panel [10]. The validity of the method is demonstrated by running a cohort of healthy newborn screening samples along with 14 confirmed galactosaemia positive samples. Furthermore, a set of 10 samples that had tested positive for other metabolic disorders in the expanded newborn screening panel including tyrosinaemia type 1 were run as well. This was done to ensure that the addition of GAL-1-P was not detrimental to the screening for other diseases. Furthermore, the presence of the milder Duarte variant was also investigated by genotyping.

## 2. Materials and Methods

### 2.1. Samples

All samples were dried blood spot (DBS) samples from the Danish national newborn screening program. The samples had been stored at −20 °C in the Danish National Biobank prior to analysis. The normal cohort consisted of approximately 5500 samples from the months of September and October in 2013. The recommended timing of these samples were 48 to 72 h after birth. Samples that were positive for galactosaemia were identified for the period 2009–2015. All forms of galactosaemia that could be identified during this period were included. Thus, some non-classical or milder forms were also investigated. The study was conducted in accordance with the Declaration of Helsinki and the protocol complies with the Danish Ethical Committee law by not being a health research project (§2,1) but a method development study not requiring an ethical approval.

In order to ensure that the addition of GAL-1-P determination to the expanded newborn screening panel did not disrupt the screening for other diseases a number of samples that had been found positive in routine screening and subsequently clinically verified were included. These sample were positive for the following disorders: multiple carboxylase deficiency (MCD), maple syrup urinary disease (MSUD), phenylketonuria (PKU), glutaracidaemia type 1 (GA-1), tyrosinaemia type 1 (Tyr-1), arginine succinate lyase deficiency (ASL), medium chain acyl-CoA-dehydrogenase deficiency (MCAD), propionic acidaemia/methylmalonic acidaemia (PA/MMA), very long chain acyl-CoA-dehydrogenase deficiency (VLCAD) and long chain 3-hydroxy acyl-CoA-dehydrogenase deficiency (LCHAD).

### 2.2. Chemicals and Reagents

The NeoBase non-derivatized newborn screening kit and the NeoBase succinylacetone assay solution were purchased from Perkin Elmer, Waltham, MA, USA. Formic acid Ph Eur 98–100% was purchased from Merck, Darmstadt, Germany. Methanol Optima LC/MS grade was purchased from Fisher Scientific, Waltham, MA, USA. Galactose-1-phosphate ≥98% was purchase from Sigma Aldrich, Copenhagen, Denmark. Galactose-1-phosphate-13C6 98.4% was purchase from Omnicron Biochemicals, South Bend, IN, USA. The extraction solution was prepared according to NeoBase kit insert (including the succinylacetone assay solution) with the following modification. An isotopically labelled GAL-1-P was added to achieve a final solution of 0.15 mmol/L. The enzyme activity assay Neonatal GALT was purchased from Perkin Elmer.

### 2.3. TMS Method

#### 2.3.1. TMS Analytical Instrument

The analytical system, which has been in use since 2009, consisted of a Quattro Micro mass spectrometer equipped with an electrospray source. It was equipped with a waters 2777C Samples manager and a Waters 1525µ LC-pump (Waters, Bedford, MA, USA). The system was controlled by Waters MassLynx software v4.1, and analytes were quantified using the NeoLynx software module of MassLynx. Extracts were introduced to the mass spectrometer by loop injection without chromatographic separation. The eluent consisted of 80% methanol in water with 0.1% formic acid. A flow profile was employed with the following settings: from zero to 0.25 min 0.12 mL/min, 0.25 to 1.15 min 0.01 mL/min, 1.15 to 1.50 min 0.7 mL/min thereafter 0.12 mL/min. Initially the system was operated in positive mode, during which time the transitions used for classical expanded newborn screening were measured. At 1.1 min the system switched to negative mode for the remainder of the run and the transitions for GAL-1-P and GAL-1-P IS were measured (259/79 and 265/79 respectively).

#### 2.3.2. Calibrators and Controls

For the determination of galactosaemia, DBS calibrators and controls were produced in house. Stock solutions of GAL-1-P with different concentrations were prepared in 9 g/L NaCl. Venous blood from a presumably healthy donor was collected directly by a single venipuncture in BD Vacutainer K2EDTA tubes (Becton Dickinson, Plymouth, UK). All tubes were filled to 6 mL as recommended by manufacturer. After the blood draw, tubes were inverted 10 times and immediately placed in beakers with ice to repress endogenous GALT activity. Next, the beakers were suspended in a 40 kHz Branson-2510 ultrasonic unit and sonicated for 30 min to denature endogenous GALT by ultrasound cavitation. The calibrators and controls were made by combining aliquots of GAL-1-P stock solution and GALT-inactivated blood. The calibrators had the following final concentrations of added GAL-1-P: 0.0625, 0.125, 0.25, 5.0, 1.0, 2.0, 4.0 and 8.0 mmol/L. Considering the concentration range of the calibrators, two quality control levels, QC low and QC high, were selected. To generate batches of QC samples low and high, GALT-inactivated blood was spiked with GAL-1-P stock solution at two concentration levels. The concentration of GAL-1-P in low QC was 0.1 mmol/L and in the high QC 1 mmol/L. The mixtures of blood were inverted carefully and the blood portions were dispensed in 75 µL aliquots onto filter paper cards (Alhstrom 226) using positive displacement pipettor to produce DBS material. The filter paper cards were allowed to dry overnight at room temperature, before being packaged in zip-lock bags with desiccant material and stored at −20 °C until use. The expanded newborn screening relied on quantitation by direct comparison with the internal standards provided in the NeoBase kit. Blanks were unspotted filter paper discs.

#### 2.3.3. Analytical Precision and Detection Limit

Repeatability was determined by punching and extracting a control sample 12 times. Reproducibility was determined using the controls that were run throughout the study. LOD is determined as the level that was three times the level observed in the blanks. The lowest limit of quantitation (LOQ) was determined as the lowest concentration that could be determined in the linear range with a CV > 20%.

### 2.4. Sample Preparation

The samples were prepared according the protocol described in the NeoBase kit insert. The only modification was the addition of GAL-1-P internal standard. Briefly, the samples were punched in microtiter plates (3.2 mm punches) using a Panthera 9 puncher (Perkin Elmer). After punching, 100 µL of extraction solution was added to all wells and the plate was incubated at 45 °C for 45 min. The extracts were subsequently transferred to a new plate. The plate was sealed and incubated at room temperature for two hours (allowing the hydrazine reaction to take place). The plate was then placed in the autosampler and 30 µL of extract was injected.

### 2.5. Enzyme Activity Analysis

The GALT enzyme activity analysis was carried out according to the kit insert. All calibrators, controls and reagents were supplied with the kit. Samples, calibrators and controls were punched using a Panthera 9 puncher. The plates were incubated in a Perkin Elmer TriNEST 1296-0050 Microplate Incubator and Shaker. Enzyme activity was determined by measuring florescence using a Victor plate reader from Perkin Elmer. Enzyme activity was measured as U/g HB.

### 2.6. Genetic Analyses

Routine genotyping for the Duarte variant (NM_000155.3:c.-119_-116del) and the t common galactosemia mutation c.563A>G, *p.Gln188Arg* (NM_000155.3:) was performed at Department of Clinical Genetics, Copenhagen University Hospital, Copenhagen, Denmark. Primers and PCR conditions are available upon request.

## 3. Results

### 3.1. TMS Method

#### Analytical Performance of the TMS Method

A linear response was observed across the calibration range (0.0625 to 8 mmol/L). The LOD was estimated to be at 0.001 mmol/L. The LOQ was found to be 0.001 mmol/L. Repeatability and reproducibility were 9% and 11% respectably.

### 3.2. Screening Performance of the TMS Method

#### 3.2.1. Normal Samples

**Figure 1:**
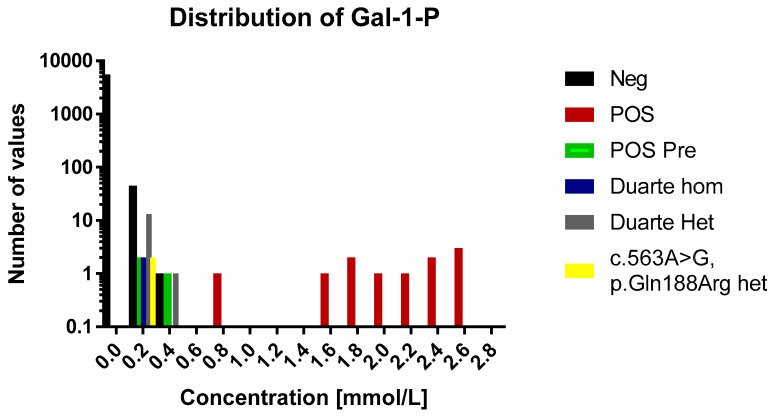
A histogram over the distribution of GAL-1-P results of the study. The histogram is divided into 0.2 mmol/L bins. Each sample category is signified by a separate color. For the sake of easy comparison the y axis is logarithmic in scale. Legend: Neg, negative for galactosaemia; POS, positive for galactosaemia; POS Pre, positive for galactosaemia diagnosed before birth; Duarte Hom, homozygote for Duarte mutation; Duarte Het, heterozygote for Duarte mutation; c. 563A>G, p.Gln 188Arg het, heterozygpte for classical galactosaemia.

**Figure 2:**
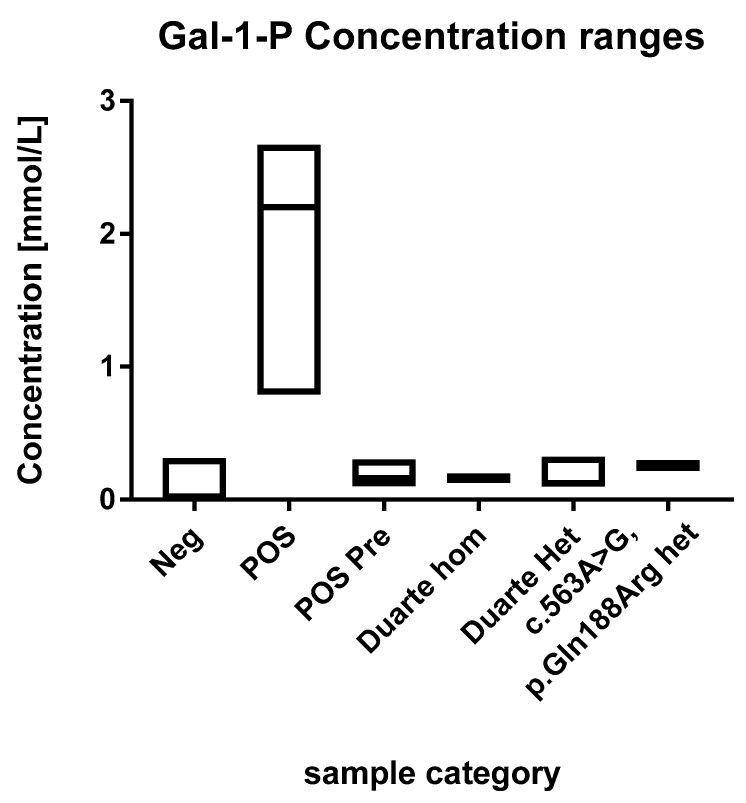
Ranges of the GAL-1-P concentrations. Each box describes the full range of the sample categories. The median values are depicted with a line within the boxes.

In the normal cohort the average concentration was 0.007 mmol/L (range 0.0005–0.31 mmol/L, *n* = 5589). The distribution of concentrations of GAL-1-P is shown in Figure 1 and Figure 2. For 83% of the samples the signal was below the LOD (0.001 mmol/L) and were assigned the concentration 0.0005 mmol/L. For samples in the range between the LOD and LOQ the calculated concentrations were used in the distribution figure.

#### 3.2.2. Galactosaemia Positive Samples

The concentrations of GAL-1-P in the positive samples were in the range of 0.1 to 2.7 mmol/L (Figure 2). These are all above the 99th percentile of the normal samples. Eleven of the fourteen positive samples were above 0.5 mmol/L which were thus well above the highest observed levels in normal samples. The three positive samples that were below 0.5 mmol/L had been diagnosed prenatally and treatment was initiated immediately after birth.

### 3.3. Duarte Variants and Galactosaemia Mutations

The 60 normal samples with the highest levels of GAL-1-P were investigated for the presence of the Duarte variant and the presence of the common galactosemia mutation (*p.Gln188Arg*) (carrier frequency 1/200). Out of these 60, two were found to be heterozygotes for the Duarte variant and 14 were homozygotes. The GAL-1-P concentrations for the Duarte homozygotes were 0.15 and 0.17 mmol/L. The heterozygotes were in the range of 0.1 to 0.32. All of samples harboring the Duarte variant were above the 99th percentile however, no separation between homozygotes and heterozygotes were observed No undetected galactosaemia cases were observed. Compound heterozygotes with the Duarte/galactosaemia combination could cause false positives when using the GAL-1-P method. However, no compound heterozygotes cases were observed in the cohort so the effect of this genetic variant has not be evaluated. Two heterozygotes for the common galactosemia mutation (*p.Gln188Arg*) were observed in the normal samples. They were clearly elevated with GAL-1-P concentrations of 0.25 and 0.26 mmol/L. The characteristics of the various sample categories are shown in Figure 2.

### 3.4. Expanded Newborn Screening Positive Samples

All ten samples positive for different metabolic disorders in the expanded newborn screening program were identified as positive in our modified method (Table A1). This demonstrates that the addition of GAL-1-P to the panel is not detrimental to screening panel.

### 3.5. Enzyme Activity Results

**Figure 3:**
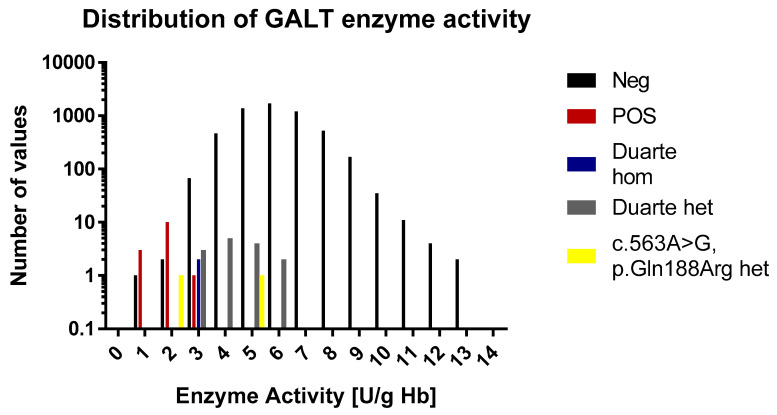
A histogram over the distribution of GALT enzyme activity results of the study. The histogram is divided into 0.2 mmol/L bins. Each sample category is signified by a separate color. For the sake of easy comparison the y axis is logarithmic in scale.

**Figure 4:**
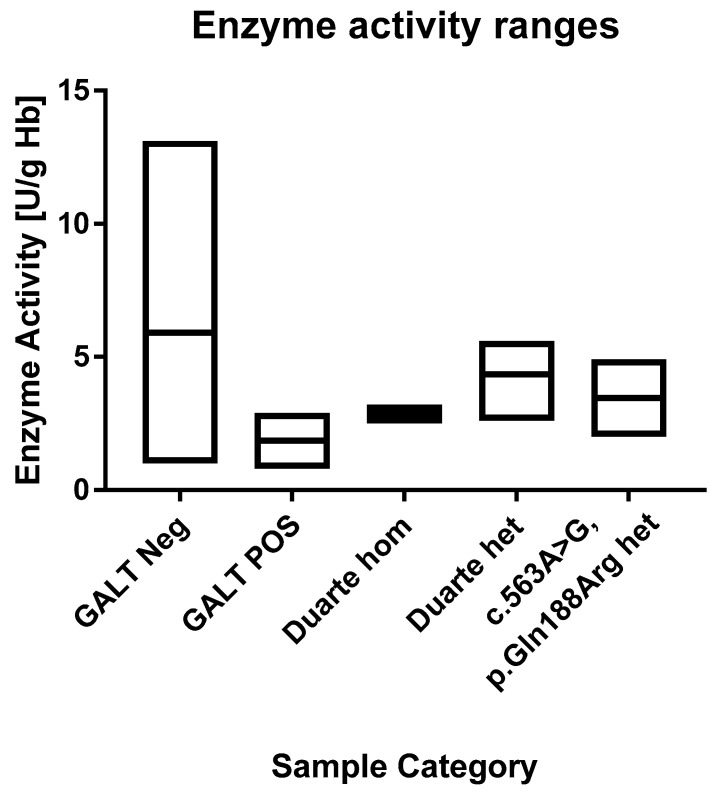
Ranges of the GALT enzyme activities. Each box describes the full range of the sample categories. The median values are depicted with a line within the boxes.

In the normal cohort the average GALT enzyme activity was 6.0 U/g Hb Hb (range 1.0 to 20.5 U/g Hb). The 1st and 99th percentile were 3.4 and 9.4 U/g Hb, respectively. This matches well with the ranges that are described in the assay’s kit inset that describes median values of 6.5 to 8.0 and a range of 3.5 to 10 U/g Hb. This indicates that very little if any depletion of enzyme activity had taken place during storage. The positive samples had an average value of 1.9 U/g Hb (range 0.8–2.9 U/g Hb). All of the positive samples are in the lowest 1th percentile. The enzyme activity values of the samples are shown in Figure 3 and Figure 4. According to the kit insert, samples ≤2.5 U/g Hb are considered to be positive and samples in the range 2.5–3.5 U/g Hb are possible positive. All positive samples except one were found to be at or below the 2.5 U/g Hb cut-off. There was one positive sample that had an activity of 2.9 U/g Hb, and thus categorized as possible positive. Among the normal samples, there were three samples that were below 2.4 U/g Hb and 87 that were below 3.5 U/g Hb.

For the 60 samples that were genotyped, the activity levels were in the range of 2.5 to 5.6. One sample (a Duarte homozygote) was 2.5 and thus would be considered a false positive and four samples were in the range of possible positive (3 heterozygotes and one homozygote). One of the normal samples that was below the 2.4 U/g Hb cut-off was found to be heterozygous for the *p.Gln188Arg* mutation.

### 3.6. Comparison of the Methods

All of the known galactosaemia samples were positive using the TMS method. These samples were positive using the enzyme activity assay as well. In the TMS the vast majority of the samples were below the LOQ and there was a clear separation between cases and controls. The enzyme assay demonstrated a bell shaped distribution with a median value of 5.9 U/g Hb. The GALT activity of the galactosaemia samples was at the low end of the distribution, but was not clearly separated from the normal samples.

## 4. Discussion

We present a TMS method for screening for galactosaemia in newborn screening samples. It is rapid, simple, and highly specific. Furthermore it can be multiplexed with expanded newborn screening. Thus, it can easily be integrated into current screening protocols. Samples from patients with classical galactosaemia were clearly elevated well above the highest normal samples. The only positives that overlapped with normal samples were samples from patients that were diagnosed before birth and were subsequently in treatment at the time of sampling. However, these samples were still clearly elevated and were above the top 1 percentile.

The method allows for rapid and efficient detection of galactosaemia in newborn screening samples. It is also more specific than previous TMS based screening methods because it measures GAL-1-P rather than HMP. HMPs are isobaric compounds that also share common fragmentation patterns. Therefore, it is not possible to differentiate between various HPMs based on mass spectrometry alone. Therefore, a mild hydrazine solution is added to the extraction solution. Hydrazine is highly reactive to keto groups. The keto group in GAL-1-P is protected from reaction by having a substitution in the 1 position. On the other hand, the keto groups of the other HMPs that are present in blood (glucose-6-phosphate (GLU-6-P) and fructose-6-phophate (FRU-6-P)) are not protected since they are not substituted in the 1 position but rather in the 6 position. During the incubation with hydrazine GLU-6-P and FRU-6-P are depleted as they react with the hydrazine. GAL-1-P remains unaffected, which makes it possible to specifically determine GAL-1-P without the need for chromatographic separation. Thus, the method is highly specific while being fully integrated into a standard expanded newborn screening panel.

The addition of hydrazine might cause reason for concern with regards to other compounds in the multiplexed panel. However, it is in fact common practice already in many newborn screening programs. During the past 10 years, Tyrosinaemia type 1 has been included into newborn screening panels worldwide [11,12]. The biomarker for this disease is succinylacetone. Succinylacetone reacts rapidly with proteins and is subsequently covalently bound. In order to liberate the covalently bound compound the samples are incubated with a mild hydrazine solution which causes the release of 3-(5-methyl-1H-pyrazol-3-yl) propanoic acid. Elevated levels of this compound are subsequently used to detect the disease. Thus, the addition of hydrazine is commonly used in newborn screening labs worldwide and does not affect the performance of the expanded screening panel.

When comparing the TMS method to the GALT enzyme assay test, our data indicate that the TMS method is better at differentiating between positive and normal samples. Screening performance aside, the TMS method offers several advantages over the enzyme assay test. Multiplexing saves one punch, integrates the analysis in the existing workflow and calls for less reagents.

Due to the clear differentiation between galactosaemia positive and normal samples it would be possible to set a cut-off that would give the TMS method an excellent positive predictive value (PPV) in a one-tier approach. Considering the data in this study, if a cut-off of 0.5 mmol/L is employed, the method would have a PPV of 100%. Known positive patients that are on diet from birth may not be detected using this cut-off. However there are several other metabolic disorders included in newborn screening programs that cannot be detected if treatment has been initiated before the sample is taken.

In a previous study [9], Jensen et al. demonstrated that quantitation of galactose-1-phosphate together with other hexose mono-phosphates may serve as biomarker for galactosaemia in newborn screening samples. At the time of that study, the newborn screening samples were taken between day 4 and 8 after birth. According to that study, exposure to milk for a minimum of three days would be necessary to create a glucose load that would result in GAL-1-P levels that are sufficiently elevated to cause a clearly elevated hexose mono-phosphate level. The samples used in the present study were taken on the second or third day of life which would be too early according to the previous study. However, we find that the GAL-1-P levels are clearly elevated in the galactosaemia positive samples despite the early sampling time. This is probably due to endogenous production of galactose [5,13,14]. Even the samples that were treated from birth with a galactose-restricted diet were clearly elevated and were in the top 1th percentile. In the method of Jensen et al., total HMP was measured which made it necessary for the GAL-1-P level to be clearly above the background level of glucose-6-phosphate for it to be positive, whereas in our method the endogenous galactose production is sufficient to induce clearly elevated GAL-1-P levels in positive patients. This is reflected in the clearly higher cut-off that they proposed. Their 1.2 mmol/L as compared to the 0.5 mmol/L that we have found suitable.

According to previous studies, one Duarte allele has 75% enzyme activity, homozygotes have 50% activity and compound heterozygotes (Duarte/Galactosaemia) have 25% activity [15]. The Duarte variant is considered to be a biochemically mild form of the disease but the penetrance is highly debated. Homozygous individuals are generally considered to be healthy and are thus not a target for newborn screening. However, due to the fact that they have reduced enzymatic activity there is a risk that they will show up as false positives. The allele frequency of the Duarte variant is reported to be 11% in the European population [16]. In the top 60 samples the frequency was 30%. Thus, individuals carrying the Duarte variant were clearly enriched in the samples with the highest levels of GAL-1-P. However, for the presented GAL-1-P method there was a clear separation between galactosemia positives and these genetic variants. For the GALT enzyme assay test, these genetic variants presented a risk for causing false positives.

The use of polarity switching in mass spectrometry is often grounds for concern. There is concern that polarity switching can put a strain on the instrument and cause a loss of performance in the long term. We found that continuous switching (switching between each scan) causes a loss in sensitivity. However, this was not necessary. Rather the polarity was switched once during each run. Only transitions for GAL-1-P and its internal standard were acquired in negative mode so only a short period of time is needed to obtain a suitable number of data points. We also observed a stabilizing effect of polarity switching where the variation of internal standard intensities was considerably smaller than when running the screening in positive mode only.

The main strength of this study is that the method has successfully been applied to 5500 biobanked samples from the Danish newborn screening cohort. The samples represent approximately all samples from one month. Furthermore, we were able to enrich the sample cohort with 14 known cases of galactosaemia. We can demonstrate that our method is robust and is a feasible routine method. A weakness to this study is that it was carried out on stored samples. There is a potential of sample degradation during storage. The normal samples had been stored at –20 °C for four years prior to analysis, and during this time both GAL-1-P and GALT enzyme activity could be degraded. The positive samples had been stored for up to eight years with a potential for further sample degradation. However, both methods were able to discriminate between normal and positive samples in a predictable manner. To truly test the method’s performance in newborn screening a prospective study would be necessary.

## 5. Conclusions

We present a method for newborn screening for galactosaemia that can easily be multiplexed into a standard expanded newborn screening TMS panel. The method is clearly superior to the commonly used GALT enzyme activity assay with regards to specificity.

## 6. Patents

“Method of diagnosing galactosemia in neonatal screening”. EP 2926139 (pending), US 9,410,960 (granted), BR 112015012773-8 (pending), CN 2013-80060296X (granted), CA 2,892,926 (pending), IN 5584/DELNP/2015 (pending) and JP 2015-544362 (pending). “Quantative analysis of hexose-monophosphates from biological samples”, EP 1311847 (validated in AT, BE, CH, DK, DE, GB, PL, ES, FR, FI, GR, IE, IT, NL, PT, SE, TR), JP4993155 (granted).

## Appendix A

**Table A1:** Results of samples with other disorder that we run using the TMS method.

Disorder	Status
MCD	confirmed
MSUD	confirmed
PKU	confirmed
GA-1	confirmed
Tyr-1	confirmed
ASL	confirmed
MCAD	confirmed
PA/MMA	confirmed
VLCAD	confirmed
LCHAD	confirmed
